# Illness Perception of Patients with Functional Gastrointestinal Disorders

**DOI:** 10.3389/fpsyt.2018.00122

**Published:** 2018-04-12

**Authors:** Na-na Xiong, Jing Wei, Mei-yun Ke, Xia Hong, Tao Li, Li-ming Zhu, Yue Sha, Jing Jiang, Felix Fischer

**Affiliations:** ^1^Department of Psychological Medicine, Peking Union Medical College Hospital, Chinese Academy of Medical Sciences & Peking Union Medical College, Beijing, China; ^2^Department of Gastroenterology, Peking Union Medical College Hospital, Chinese Academy of Medical Sciences & Peking Union Medical College, Beijing, China; ^3^Department of Internal Medicine, Peking Union Medical College Hospital, Chinese Academy of Medical Sciences & Peking Union Medical College, Beijing, China; ^4^Medical Clinic for Internal Medicine, Department of Psychosomatic Medicine, Charité – Universitätsmedizin Berlin, Berlin, Germany

**Keywords:** functional gastrointestinal disorders, illness perceptions, alexithymia, non-gastrointestinal symptoms, China

## Abstract

**Objective:**

To investigate the illness perception characteristics of Chinese patients with functional gastrointestinal disorders (FGID), and the mediating role between symptoms, psychopathology, and clinical outcomes.

**Methods:**

Six illness groups from four outpatient departments of a general hospital in China were recruited, including the FGID patient group. The modified and validated Chinese version of the illness perception questionnaire-revised was utilized, which contained three sections: symptom identity, illness representation, and causes. The 12-item short-form health survey was utilized to reflect the physical and mental health-related quality of life (HRQoL). The Toronto alexithymia scale was used to measure the severity of alexithymia. Additional behavioral outcome about the frequency of doctor visits in the past 12 months was measured. Pathway analyses with multiple-group comparisons were conducted to test the mediating role of illness perception.

**Results:**

Overall, 600 patients were recruited. The illness perceptions of FGID patients were characterized as with broad non-gastrointestinal symptoms (6.8 ± 4.2), a negative illness representation (more chronic course, worse consequences, lower personal and treatment control, lower illness coherence, and heavier emotional distress), and high numbers of psychological and culture-specific attributions. Fit indices of the three hypothesized path models (for physical and mental HRQoL and doctor-visit frequency, respectively) supported the mediating role of illness perceptions. For example, the severity of alexithymia and non-gastrointestinal symptoms had significant negative effect on mental quality of life through both direct (standardized effect: −0.085 and −0.233) and indirect (standardized effect: −0.045 and −0.231) influence *via* subscales of consequences, emotional representation, and psychological and risk factor attributions. Multi-group confirmatory factor analysis showed similar psychometric properties for FGID patients and the other disease group.

**Conclusion:**

The management of FGID patients should take into consideration dysfunctional illness perceptions, non-gastrointestinal symptoms, and emotion regulation.

## Introduction

Functional gastrointestinal disorders (FGIDs), defined as disorders of gut–brain interaction, refer to multiple chronic or recurrent gastrointestinal symptoms without any evident organic or structural abnormalities as revealed by regular clinical and laboratory investigations (1). Among them, irritable bowel syndrome (IBS) and functional dyspepsia (FD) are the most common.

Recent research has improved our theoretical understanding of FGIDs. However, the clinical management of such disorders still faces challenges and frustrations. One neglected reason might be physicians’ lack of knowledge of patients’ perceptions about their illnesses and their demands. Developed from the self-regulation model of Leventhal and colleagues (2), illness perception refers to the model one creates to make sense of and respond to one’s symptoms and the related problems, which mainly includes their own ideas about the identity, time line, consequences, controllability, and causes of their illness. Illness perceptions can vary greatly from one to another, even in those with same symptoms. Previous studies suggested that gastroenterologists and patients held different perceptions toward FGID (3–5). For example, patients believed that their problems were more severe and more disabling than perceived by the physician. In addition, illness perceptions have been shown to influence adherence to treatment advice and health outcomes across a range of illnesses (6–8). Among them, one study found that positive change in patients’ illness perceptions improved IBS symptoms and disability through the cognitive behavioral therapy intervention (9).

Even though FGIDs are not a group of life-threatening diseases, patients’ health-related quality of life (HRQoL) was comparably or even more impaired when compared with those with chronic organ diseases (10–12). In addition, FGID was associated with increased outpatient services, surgeries, and health-care costs (13). It has also been reported that the HRQoL not only had a direct relationship with the severity of IBS symptoms but also an indirect relationship *via* the patients’ cognitive and emotional representations of their illness, which meant mediated by the patients’ illness perceptions (14). However, only gastrointestinal symptoms have been taken into account, and no help-seeking behavioral outcomes have been included. Recent studies suggested that non-gastrointestinal comorbidities were also common in FGID patients, such as sleeping or sexual problems (13, 15, 16). In addition, alexithymia, which refers to persons having difficulty in identifying and expressing their emotions, and having the tendency to talk about their somatic discomforts, instead of emotional conflicts (17), is considered to be a trait and a vulnerability factor for the occurrence, severity, and treatment outcomes of FGID (18–20). Therefore, our hypothesis was that the severity of non-gastrointestinal symptoms and alexithymia also had an influence on clinical outcomes, including HRQoL and the behavioral variable of doctor visits. Based on the principle of cognitive behavioral theory, we further hypothesized that cognition of the illness could have a mediating role among the above pathways.

In particular, unlike western countries, FD was the most common subtype of FGIDs in China (21). Chinese also place vital importance on gastrointestinal function, as the old saying goes, “food is the first necessity of the people.” In comparison with westerners, illness perception of Chinese patients was at least additionally influenced by superstitious beliefs about devil spirits and theories from Traditional Chinese Medicine (TCM) (22).

Thus, this study aimed (1) to describe the illness perception characteristics of Chinese patients with FGID and (2) to explore the mediating role of illness perception among gastrointestinal and non-gastrointestinal symptoms, alexithymia, and clinical outcomes among them.

## Materials and Methods

### Study Design and Procedure

This cross-sectional study was conducted in the outpatient departments of a general hospital in Beijing, China between May 2014 and July 2015. Two groups of patients were recruited from the gastroenterology department: those with FGID and those with peptic ulcer or reflux esophagitis (PU/RE). Patients who fulfilled the Rome III diagnostic criteria of any type of FGIDs and did not have any organic or structural abnormalities to account for the gastrointestinal symptoms were assigned to the FGID group. The diagnoses of patients with PU/RE were made according to clinical symptoms (like abdominal pain or heartburn) and ulcers confirmed by upper gastrointestinal endoscopy within a month. Another two groups of patients with major depressive disorder (MDD) and somatic symptom disorder (SSD) were recruited to represent those with prominent psychological and somatic distress, respectively. To be included, patients had to fulfill the diagnostic criteria according to the *Diagnostic and Statistical Manual of Mental Disorders, fifth edition* (DSM-5) (23). Another two groups of general patients from the TCM and the general internal medicine (GIM) departments were recruited to represent the general outpatient population within both the traditional and western medicine model, regardless of their diagnoses.

All participants had to be 18 years or older, seeking treatment voluntarily for their own problems, and able to read and sign the informed consent form. Participants were excluded if they had language barriers, limited writing skills, cognitive impairment/brain disorders/dementia, psychosis, or acute suicidal tendencies.

On randomly assigned screening days, all eligible patients who attended those clinics were consecutively informed and invited to participate the study by research assistants. All patients, including those who declined participation, were registered with their respective reasons.

### Assessment Instruments

#### The Chinese Version of the IPQ-R

The illness perception questionnaire (IPQ) (24) and its revised version (IPQ-R) (25) are the most commonly used instruments to assess views of illnesses. The IPQ-R comprises three components: symptom identity, illness representations, and causes of the illness. Even though the Chinese version of the IPQ-R was available and applied to patients with acute myocardial infarction (26, 27), the factor structure was highly unstable in different populations (28). Therefore, based on the current version at the official website (29), a slightly cultural modification and adaptation was made in the first phase of this study. The internal consistency, test–retest reliability, structure validity, known-group validity and criterion validity have been proved to be satisfactory (30).

After the adaptation, 28 symptoms were included in the symptom identity section to cover common complaints in general hospital outpatients. Each was associated with two yes/no answers to measure whether or not the patient experienced the symptom and whether or not they believe it was related to their illness. Symptoms were divided into two dimensions for comparison: gastrointestinal symptoms (including 10 symptoms derived from the digestive tract) and non-gastrointestinal symptoms (concerning 18 symptoms in the cardiopulmonary system, bodily pain, sleeping problems, mood disorders, and other sensory discomforts).

All 38 items in the illness representation component were retained without adaptation, since its psychometric properties have been shown to be consistent in various settings and illness groups. Scored on a Likert scale of 1 (strongly disagree) to 5 (strongly agree), a seven-factor structure was proposed to reflect how patients experience their illnesses from the following seven dimensions (25): acute/chronic time line, cyclical time line, consequences, personal control, treatment control, illness coherence, and emotional representations. This seven-factor structure was proved to be satisfactory in this sample (30).

Concerning the causal subscale, five cultural-specific illness attributions were added according to previous studies (31), including “endocrine disorders,” “puerperium illness (only for women),” and attributions according to TCM theories. The five-point Likert scale was also used for the final 23 items. Four factors were generated with the exploratory factor analysis, which were named the psychological factors (e.g., “stress or worry,” “my mental attitude,” etc.), cultural-specific factors (e.g., “diet or eating habits,” TCM theories, etc.), risk factors (e.g., “smoking” and “alcohol”), and accidental attributions (e.g., “poor medical care in the past” and “chance or bad luck”) (30).

#### The 9-Item Patient Health Questionnaire (PHQ-9)

The PHQ-9 was used to measure the depression severity, with nine items derived from the diagnostic criteria of major depression disorder according to the DSM-IV. To screen for MDD in the general population, a cutoff value of 10 has been validated (32). It was also proved to be reliable and valid to detect major depression in Chinese patients with multiple somatic symptoms (33).

#### The 7-Item Anxiety Scale (GAD-7)

The seven items was used to measure the severity of generalized anxiety according to the diagnostic criteria of DSM-IV. A meta-analysis suggested that the GAD-7 had good operating characteristics for detecting generalized anxiety with an optimal cutoff point of 10. Using this cutoff point, the GAD-7 has demonstrated good reliability and validity in screening anxiety disorders in Chinese general hospital outpatients (34).

#### The 12-Item Short-Form Health Survey (SF-12)

The 12 items captures practical, reliable, and valid information on HRQoL in the previous 4 weeks (35), which produces a physical composite score (PCS) and a mental composite score (MCS). The SF-12 has been demonstrated to be reliable and valid for use with the Chinese population (36), with higher scores indicating subjective better well-being. The mean standard PCS and MCS scores of the Hong Kong subjects were also found to be similar to the US population mean of 50 (36).

#### The Toronto Alexithymia Scale (TAS)

The TAS is a self-rated questionnaire designed to reflect the severity of alexithymia (37). The terminology “alexithymia” was first raised to describe patients with psychosomatic illnesses who often have difficulties in expressing and distinguishing their emotions. In the TAS, 26 items were rated on a five points Likert scale, with higher scores indicated more severe alexithymia. The Chinese TAS has demonstrated sound psychometric performance (38).

Additional questions were asked about the symptom duration (for those with more than one symptom, it was defined as the duration of the symptom brought them to the doctor today) and the frequency of doctor visits for the current symptoms or illnesses in the past 12 months (1 = none; 2 = one or two times; 3 = 3–10 times; 4 = 11–20 times; 5 = more than 20 times).

### Statistical Procedures

Descriptive data are presented as the means and SDs for continuous variables and absolute and relative frequencies for categorical variables. To test whether the sociodemographic features among six groups differed, one-way analysis of variance was used for continuous data with normal distribution, the Kruskal–Wallis test was used for continuous data with skew distribution, and the χ^2^ test was used for categorical variables. To control the potential confounding effect of age, gender, and educational level, the analysis of covariance was conducted in the comparison of clinical features, including the illness perception and psycho-behavioral variables. Pearson’s correlation analysis was performed to assess the association between two variables. For each variable of interest, no missing value was detected. A *p*-value of less than 0.05 (two-tailed) was considered significant.

To test the mediating role of illness perceptions, first, multiple linear regressions were conducted among the whole sample to find potential significant predictors for the mediators (subscales of the illness representation and the causal components) among latent variables (the total TAS score and the number of gastrointestinal and non-gastrointestinal symptoms), and then predictors for outcomes (HRQoL and doctor-visit frequency) from both the latent variables and mediators. Second, three path models were constructed and tested within the whole sample with the bootstrapped maximum likelihood method (for continuous data with normal distribution, i.e., HRQoL) and the asymptotically distribution-free method (for categorical data, i.e., doctor-visit frequency). Pathways with a non-significant contribution (*p* > 0.05) were deleted. Reasonable correlations were allowed according to the modification indices to further improve the model fit. Model fit was examined using Chi-square difference tests and inspection of the Root Mean Square Error of Approximation (RMSEA) and the Comparative Fit Index (CFI). A value of 0.05 or less for RMSEA was considered to be very good, while 0.05–0.08 was acceptable (39). A value of 0.95 or greater for CFI was considered to be adequate (40). Finally, to test whether these pathways and effects were equivalent in FGID patients, the overall sample was split into the FGID subgroup and the non-FGID subgroup for the multiple-group analysis. All coefficients were constrained to be equal across the subgroups, and the subsequent model fit indices for the FGID subgroup were examined.

The sample size was determined by the sample needed to adapt and validate the Chinese version of IPQ-R. Statistical analyses were performed with IBM SPSS Statistics 20.0 and AMOS 21.0.

## Results

### Characteristics of Participants

Among 679 patients who have been contacted, 600 patients (88.4%) were included in the study. The main reasons for not enrolling were lack of time (*n* = 48, 60.8%), lack of interest in the study (*n* = 21, 26.6%), or other reasons (*n* = 10, 12.7%) such as difficulty with reading. As shown in Table 1, the proportions of male and elderly patients in the PU/RE group were the highest, while the percentages of unemployed and less educated patients were highest in the FGID group. FGID patients also had the longest illness duration.

**Table 1:** Sociodemographic and clinical characteristics of six illness groups of participants (*n* = 600).

Illness groups	*n*	Gender (female %)	Age (M ± SD)	Living area (city %)	Marital status (married %)	Occupation (unemployed %)	Education level (≥university %)	Illness duration (months)
FGID	102	61.8	43.7 ± 14.0	80.4	78.4	46.1	37.3	24.0 (11.5, 60.0)
PU/RE	95	37.9^a^	49.8 ± 12.9^a^	90.5	78.9	43.2	33.7	10.0 (3.0, 24.0)
MDD	100	61.9	42.7 ± 15.8	88.7	61.9	35.1	60.8^a^	11.5 (6.0, 19.5)
SSD	101	58.4	41.7 ± 14.8	80.2	71.3	33.7	49.5	18.0 (7.0, 36.0)
TCM	102	60.8	39.9 ± 13.6	81.4	68.6	24.5^a^	61.4^a^	18.0 (6.0, 36.0)
GIM	100	58.0	41.9 ± 16.2	80.0	69.0	38.0	45.0	12.0 (3.0, 36.0)
Total	600	56.6	43.2 ± 14.9	83.4	71.4	36.7	48.0	12.0 (6.0, 36.0)
χ^2^*/F*		16.7	5.3	8.0	10.1	12.7	26.6	31.3
*p*-Value		**0.005**	**<0.001**	0.157	0.072	**0.027**	**<0.001**	**<0.001**

*χ^2^/F values and p values of comparisons with other sociodemographic characteristics have been controlled for age and gender*.

*FGID, functional gastrointestinal disorders; GIM, general internal medicine; MDD, major depressive disorder; PU/RE, peptic ulcer/reflux esophagitis; SSD, somatic symptom disorders; TCM, traditional Chinese medicine*.

*^a^Indicated values that contributed most to the statistically significant difference*.

*The bold font indicated p-value **<**0.05*.

The specific clinical diagnoses and their percentages within each patient group were noted and provided in Tables S1–S4 in Supplementary Material. In the FGID group, the most common diagnoses were FD (31.4%), FGID (for those with overlap subtypes of FGIDs, 26.5%), and functional constipation (18.6%), while the percentage of patients with IBS alone was only 3.9%. In the PU/RE group, 41.1% patients had peptic ulcer, 58.9% had reflux esophagitis, and 3.2% had both. Of note, 10.8% patients in TCM department and 12.0% patients in GIM department had gastrointestinal complaints, but their medical diagnoses was unregistered due to our study design.

### Characteristics of the Illness Perception of FGID Patients

Besides the largest number of gastrointestinal symptoms, FGID patients also experienced broad non-gastrointestinal symptoms (6.8 ± 4.2), which was significantly more than those of PU/RE patients and patients from GIM, but still less than those of MDD and SSD patients (see Table 2).

**Table 2:** Characteristics of the illness perception of six illness groups of participants (*n* = 600).

Illness groups	*n*	Symptoms identity		Illness representation							Causes			
		
		GI	Extra-GI	Time line	Cyclical	Consequences	Personal control	Treatment control	Illness coherence	Emotional representation	Psychological attribution	Culture-specific attribution	Risk factors	Accident
FGID	102	5.4 ± 2.4^a^	6.8 ± 4.2^b^	15.4 ± 4.1	12.7 ± 2.3	18.4 ± 4.7^b^	19.1 ± 3.8^b^	17.6 ± 2.6^b^	14.0 ± 3.7^b^	20.2 ± 4.9^a^	17.8 ± 4.3^b^	19.4 ± 3.5^a^	4.1 ± 1.4^b^	7.5 ± 2.0
PU/RE	95	4.2 ± 2.0^b^	4.6 ± 3.7^c^	14.0 ± 3.4^b^	12.8 ± 2.3	16.9 ± 4.3^c^	21.1 ± 3.3^a^	18.7 ± 2.3^a^	15.8 ± 3.3^a^	17.9 ± 4.8^b^	16.7 ± 3.7^c^	19.5 ± 3.1^a^	5.1 ± 1.7^a^	7.2 ± 1.5^b^
MDD	100	4.4 ± 2.8^b^	10.5 ± 4.0^a^	15.4 ± 4.0	12.1 ± 2.5	19.3 ± 4.5^a^	20.1 ± 3.2	18.2 ± 2.3^a^	15.5 ± 3.3^a^	20.0 ± 4.9^a^	20.5 ± 4.1^a^	16.7 ± 4.4^b^	4.0 ± 1.2^b^	7.1 ± 2.0^b^
SSD	101	4.2 ± 2.7^b^	9.7 ± 3.8^a^	16.7 ± 3.8^a^	12.4 ± 2.6	19.6 ± 4.6^a^	19.7 ± 3.6	16.5 ± 2.8^c^	13.8 ± 3.9^b^	21.1 ± 4.2^a^	18.8 ± 3.9^b^	17.6 ± 3.4^b^	3.8 ± 1.2^b^	8.3 ± 1.9^a^
TCM	102	3.6 ± 2.8^b^	6.7 ± 4.3^b^	17.1 ± 4.5^a^	12.0 ± 2.8	16.1 ± 5.0^c^	20.7 ± 3.4^a^	17.7 ± 2.8^b^	15.3 ± 4.0	17.9 ± 4.8^b^	16.4 ± 4.4^c^	20.1 ± 3.9^a^	4.2 ± 1.9^b^	7.9 ± 2.1
GIM	100	2.2 ± 2.2^c^	5.3 ± 3.8^c^	16.3 ± 5.0^a^	12.1 ± 3.0	18.5 ± 5.5^b^	20.8 ± 4.0^a^	18.0 ± 2.8^a^	14.4 ± 4.0	18.9 ± 5.0^b^	15.0 ± 3.3^c^	17.1 ± 3.0^b^	4.4 ± 1.2^b^	7.4 ± 1.7^b^
Total	600	4.0 ± 2.7	7.3 ± 4.5	15.9 ± 4.3	12.3 ± 2.7	18.1 ± 5.0	20.2 ± 3.6	17.7 ± 2.7	14.8 ± 3.8	19.4 ± 4.9	17.5 ± 4.4	18.4 ± 3.8	4.3 ± 1.6	7.6 ± 1.9
*F* value		17.5	37.1	7.7	1.7	9.0	4.2	8.0	4.5	7.9	26.8	15.5	5.7	5.4
*p*-Value		**<0.001**	**<0.001**	**<0.001**	0.170	**<0.001**	**0.001**	**<0.001**	**<0.001**	**<0.001**	**<0.001**	**<0.001**	**<0.001**	**<0.001**

*F and p values of comparisons have been controlled for age, gender, occupation, and education level. The Bonferroni method was adopted for multiple comparisons: values with ^a^ were significant higher than values with ^b^, and values with ^b^ were significant higher than values with ^c^ in multi-group comparison*.

*FGID, functional gastrointestinal disorders; GI, gastrointestinal symptoms; GIM, general internal medicine; MDD, major depressive disorder; PU/RE, peptic ulcer/reflux esophagitis; SSD, somatic symptom disorders; TCM, traditional Chinese medicine*.

*The bold font indicated p-value **<**0.05*.

In addition, compared to PU/RE patients, FGID patients had a negative illness representation (more chronic course, worse consequences, lower personal and treatment control, lower illness coherence, and heavier emotional distress), which was similar to MDD and SSD patients. Notably, FGID patients felt they could do least to help themselves, had least comprehension of the illness and were most emotionally distressed among all six groups of participants, including patients from the GIM and the TCM department commonly with chronic organic diseases.

In terms of causes of their illnesses, the most commonly endorsed items of FGID patients were “incoordination between the spleen and the stomach/deficiency of qi and blood (according to TCM theory)” (81.4%), “stress or worry” (67.6%), and “diet or eating habits” (60.8%). In comparison, patients with FGID attributed more psychological factors than patients with PU/RE and patients from the GIM and the TCM department, but less than patients with depression. Like those in TCM department, FGID and PU/RE patients also selected a higher number of culture-specific items, but less risk factors, as the causes of their illnesses.

### Alexithymia, Psychological Distress, HRQoL, and Doctor Visits of FGID Patients

Compared with other five groups of patients, patients in the FGID group had the highest level of anxiety, and a moderate level of alexithymia, depression, mental and physical HRQoL, and doctor-visits frequency (see Table 3), which was only better than SSD patients.

**Table 3:** Alexithymia, psychological distress, health-related quality of life (HRQoL), and doctor visits in the past 12 months of six illness groups of participants (*n* = 600).

Illness groups	*n*	Alexithymia	Psychological distress		HRQoL		Doctor visits (times %)			
			
		Toronto Alexithymia Scale	Patient Health Questionnaire-9	The 7-Item Anxiety Scale	Physical composite score	Mental composite score	0–2	3–10	11–20	> 20
FGID	102	72.0 ± 8.0	8.6 ± 6.0^b^	7.2 ± 5.3^a^	43.1 ± 8.1	41.7 ± 10.2^b^	16.7	50.0	17.6	15.7
PU/RE	95	70.9 ± 5.4^b^	4.9 ± 3.6^c^	4.5 ± 3.8^b^	45.4 ± 6.4^a^	47.4 ± 8.6^a^	32.6	56.8	10.5	0
MDD	100	74.6 ± 9.0^a^	12.0 ± 7.3^a^	8.1 ± 6.3^a^	43.2 ± 8.6	35.3 ± 12.5^c^	24.5	48.0	18.4	9.2
SSD	101	74.3 ± 7.4^a^	10.4 ± 0.6.0^a^	8.1 ± 4.9^a^	40.1 ± 8.0^b^	37.4 ± 9.8^c^	11.9	37.6	26.7	23.8
TCM	102	73.4 ± 8.5	7.7 ± 4.9^b^	6.0 ± 4.6^b^	45.0 ± 8.1^a^	44.4 ± 9.9^a^	33.3	43.1	13.7	9.8
GIM	100	68.9 ± 8.0^b^	5.7 ± 5.0^c^	4.1 ± 4.3^b^	40.0 ± 9.9^b^	46.3 ± 9.7^a^	27.0	56.0	10.0	7.0
Total	600	72.4 ± 8.0	8.2 ± 6.1	6.3 ± 5.1	42.8 ± 8.5	42.1 ± 11.1	24.2	48.5	16.2	11.0
χ^2^/*F*		9.7	22.5	13.4	7.8	23.0	12.7			
*p*-Value		**<0.001**	**<0.001**	**<0.001**	**<0.001**	**<0.001**	**<0.001**			

*χ^2^/F and p values of comparisons have been controlled for age, gender, occupation, and education level. Values with ^a^ were significant higher than values with ^b^, and values with ^b^ were significant higher than values with ^c^ in multi-group comparison*.

*FGID, functional gastrointestinal disorders; GIM, general internal medicine; MDD, major depressive disorder; PU/RE, peptic ulcer/reflux esophagitis; SSD, somatic symptom disorders; TCM, traditional Chinese medicine*.

*The bold font indicated p-value **<**0.05*.

At the cutoff points of 10 for the PHQ-9 and GAD-7 to screen for major depression disorder and generalized anxiety disorder, 38.2 (39/102) and 32.4% (33/102) of patients with FGID were positive, respectively. The physical and mental quality of life of 77.5% (79/102) and 74.5% (76/102) patients with FGID fell below of 50, the mean standard PCS and MCS score for the general population. During the past 12 months, 17.6% of patients with FGID have visited doctors 11–20 times for their problems, and 15.7% of them have visited doctors more than 20 times.

The levels of alexithymia, depression, and anxiety and the frequency of doctor visits were positively correlated with the number of experienced symptoms (*r* = 0.202–0.586, *p* < 0.001) and a negative illness representation (|*r*| = 0.145–0.513, *p* < 0.001), while the correlation between the level of HRQoL and these factors was the inverse (|*r*| = 0.131–0.491, *p* < 0.001).

### The Illness Perception Mediates Between Alexithymia, Symptoms, and Outcomes

**Figure 1:**
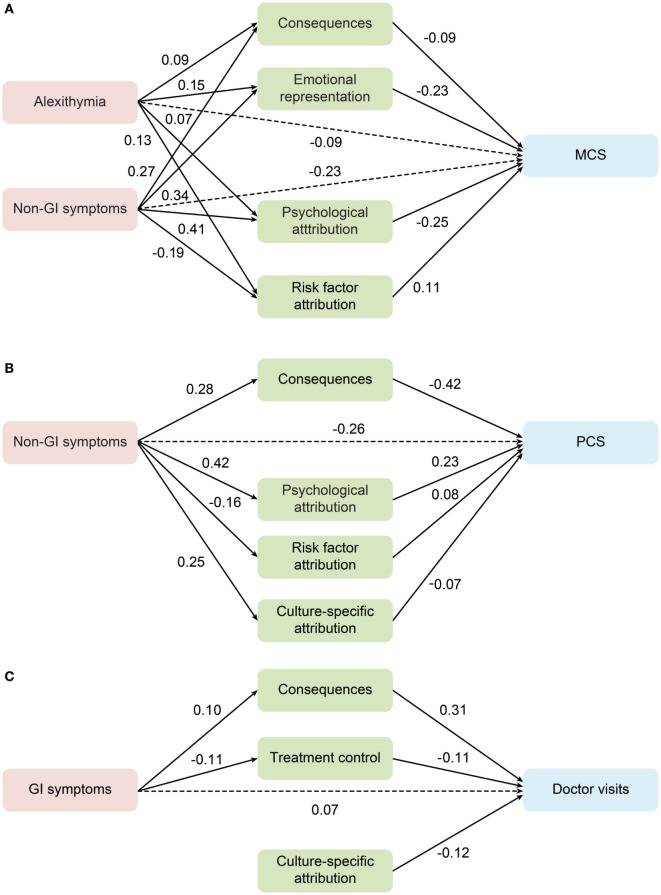
Mediation role of the illness perception between alexithymia, gastrointestinal and non-gastrointestinal symptoms, and outcomes in the functional gastrointestinal disorder group. **(A)** MCS, mental composite score; **(B)** PCS, physical composite score; **(C)** the frequency of doctor visits. Standardized estimates are shown for the significant regression paths. Residual errors are omitted from the figure.

The first analysis examined the mental dimension of HRQoL as an outcome in the whole sample. As summarized in Table S5 in Supplementary Material, the model fit indices were satisfactory. In this model, non-gastrointestinal symptom severity and alexithymia had a direct effect and indirect effect in predicting worse mental HRQoL *via* illness perception. To test whether the model applied to FGID patients specifically, further multiple-group analyses were conducted, when all coefficients were constrained to be equal across FGID patients and all others. As a result, fit indices of the model in FGID group alone were acceptable [χ^2^ (d.f.) = 45.3 (20), RMSEA (95% CI) = 0.046 (0.028–0.064), CFI = 0.975], suggesting that the model also fit patients with FGID (see Figure 1A).

The same analyses were conducted for the physical dimension of HRQoL and the frequency of doctor visits during the past 12 months in the overall population. Only the severity of non-gastrointestinal symptoms had a significantly inverse direct and indirect contribution to PCS. Model fit indices for FGID patients alone were found acceptable with the same coefficients [χ^2^ (d.f.) = 38.3 (17), RMSEA (95% CI) = 0.046 (0.026–0.065), CFI = 0.955] (see Figure 1B).

The model of doctor-visit frequency, however, only included paths from gastrointestinal symptoms through subscales of the consequence and treatment control, while risk factor attribution had a direct contribution. Model fit indices for both the overall and FGID subgroup within the multi-group comparison [χ^2^ (d.f.) = 6.6 (12), RMSEA (95% CI) = 0.000 (0.000–0.021), CFI = 1.000] were satisfactory (see Figure 1C).

The results of the pathway models can be summarized as follows: (1) models examined in the overall group were applicable for FGID patients; (2) subscales of consequences, emotional representation, and treatment control, as well as illness attributions played a key mediating role; (3) alexithymia was an independent predictor for mental HRQoL; (4) it was the number of non-gastrointestinal symptoms, instead of gastrointestinal symptoms, that predicted worse mental and physical HRQoL; and (5) only gastrointestinal symptoms had a significant direct and indirect effect on doctor-visit frequency both in the overall sample and in patients with FGID.

## Discussion

This study expands upon past research by exploring illness perception characteristics of Chinese FGID patients, as well as their mediating role between alexithymia, gastrointestinal and non-gastrointestinal symptoms, and doctor-visit frequency.

### The Illness Perceptions of Chinese FGID Patients

From the perspective of symptomology, this study adds evidence for the broadness of non-gastrointestinal symptoms in FGID patients. Actually, the phenomenon of high comorbidity with psychiatric and extra-intestinal functional disorders among FGID patients has been noticed for long (13, 15, 16). Potential mechanisms of the bidirectional communication between the brain and the gut have been proposed.

Regarding illness representations, FGID patients were negative, particularly with regard to low self-control and illness coherence and high emotional distress. The extent of limited understanding of the illness, and low belief in personal control was comparable to patients with chronic organic disease, such as epilepsy (41). Similarly, a study found that patients with IBS perceived the illness to be a chronic condition with negative consequences (42). Gastroenterologists and nurses also thought that compared with patients with IBS, those with inflammatory bowel disease (IBD) could better understand their illness (5). Actually, the sense of comprehension and control over one’s illness and oneself was craved by all patients. Even though nowadays the diagnosis of FGIDs still means an exclusive procedure of organic diseases and no diagnostic examinations or biomarkers are available, to ease such frustration, physicians need to address the diagnoses adequately in their clinical practice. The discrepancy between physicians’ and patients’ perception of FGIDs could hamper the effective establishment of a therapeutic doctor–patient relationship (3, 4, 22), while the effective changes in negative beliefs were discovered to improve the HRQoL and social adjustment in IBS patients (13).

In terms of causes of their illnesses, this study found that Chinese patients with FGID attributed more psychological factors than patients with organic diseases, but less than patients with depression. However, a previous study indicated that only physicians considered psychological causes to play a causative role in IBS and that no such difference was found in patients (4). In addition, like those in TCM department, both FGID and PU/RE patients from the gastrointestinal department selected a higher number of culture-specific items, but less risk factors such as smoking and alcohol use, as the causes of their illnesses. One of the potential explanations was the special attention to gastrointestinal function and food in Chinese culture. For example, as is stated in the *Yellow Emperor’s Internal Classics*, one of the classical books of TCM theory, “sufficient healthy-*Qi* inside the body prevents the invasion of pathogenic factors.” To a certain extent, healthy-*Qi* is referred to today as the immunity system for the Chinese. Therefore, it is comprehensible for Chinese patients with FGID to choose “altered immunity”, one culture-specific item, as one of the most common causes of their illnesses. As a matter of fact, it is intriguing that modern medicine has also demonstrated the role of intestinal microbes and mucosal immune function in the pathological mechanisms of FGIDs.

### The Mediating Role of Illness Perceptions

Until now, two studies have investigated the mediating role of illness representation among gastrointestinal symptoms and outcomes in IBS patients. One of them confirmed the mediating role of consequence and emotional representation on total HRQoL, dysphoria, interference with activity, food avoidance, social reaction, and sexuality, even though only the brief IPQ was adopted, in which each subscale of the illness representation was measured with one single item and was less reliable (14). Used the old version of the IPQ, another study showed that serious consequences and weaker control belief was associated with lower quality of life and lower satisfaction with health (43). Similar as their results, our study confirmed that alexithymia and non-gastrointestinal symptoms had negative impact on mental quality of life through the mediating of consequences, emotional distress, and psychological attribution, that non-gastrointestinal symptoms had negative influence on physical quality of life through the mediating of consequences and culture-specific attribution, and that gastrointestinal symptoms lead to more doctor visiting through the mediating of consequences and treatment control. Besides, illness perceptions were found to have an even stronger relationship with outcomes than indicators of the severity of illnesses across a range of organic diseases (44). A systematic review also provided evidence for the effectiveness of improving adherence by targeting cure/control perceptions (45). This suggested clinicians focus not only on reducing symptoms, but also on identifying and changing negative dysfunctional beliefs. For future research with FGID patients, it is advisable to also include measures like medication adherence and response rate.

### Alexithymia and Mental HRQoL

Previously, patients with FGIDs were found to have higher scores of alexithymia than the healthy population (18). According to our results, FGID patients demonstrated a moderate level of alexithymia, which was better than those with major depression and chronic somatic symptoms, but worse than those with PU/RE or other organic diseases. Furthermore, another study showed that compared with improved patients with FGID, unimproved patients had significantly higher levels of alexithymia. Thus, it was suggested to be a predictor of treatment outcomes in FGID patients (19). The pathway analyses in our results also supported the direct and indirect effect of alexithymia on mental HRQoL, but not on physical HRQoL or frequency of doctor visiting. A randomized controlled trial study showed that brief psychodynamic therapy could improve the gastrointestinal symptoms of patients with FD as well as their difficulties in identifying and describing their feelings (21). Future studies should further explore the effectiveness of targeting alexithymia in the treatment of FGIDs.

### Non-Gastrointestinal Symptoms and HRQoL

The results showed that the number of non-gastrointestinal symptoms, rather than gastrointestinal symptoms, predicted worse HRQoL. Previous studies found that unlike patients with organic gastrointestinal illnesses, the severity of gastrointestinal symptoms, as well as their improvement during treatment, played only a weak role in predicting overall health and disability among FGID patients (46). Instead, non-gastrointestinal symptoms, such as fatigue, were found to have a major effect (13). For clinicians, our results highlight the importance of recognizing and assessing the severity of non-gastrointestinal symptoms, particularly under the condition that patients have learned to only talk about “relevant” symptoms to certain doctors, likely due to the division of medical specialties.

### Gastrointestinal Symptoms and Doctor-Visit Frequency

Patients experienced their bodies and the outside world differently from common life, and help-seeking behaviors are endeavors to fight against the devastation of life worlds (47). This study was the first to find that gastrointestinal symptoms could independently predict doctor-visit frequency not only in FGID patients but also in the overall sample. Similarly, based on the second Dutch National Survey of general practice, patients with upper gastrointestinal symptoms visited their primary care physicians twice as frequently as controls (48). However, studies conducted in the normal elderly population (49) and Japanese general population (50) found that even though abdominal symptoms were frequent, only a minority contacted doctors. Therefore, it was speculated that the subjects’ concept of the symptom as a health problem or not contributed to whether they visited a doctor, particularly with regard to consequences and treatment control. As discussed above, the emphasis the Chinese place on food and gastrointestinal function might contribute to this phenomenon. More studies are needed to examine this relationship in different populations.

### Limitations

This study has several limitations. First, the study was cross-sectional, so the causal effect of our model could not be established. It is possible that the interrelationship is the reverse, or that there is a common cause to both the poor outcomes and the negative illness representations. Therefore, prospective and interventional studies are needed to explore the relationships among the variables of psychological disposition, illness perception, and clinical outcomes. Second, this study did not include measurements of disease activity and coping strategies, which have been suggested to have a potential interrelationship with illness perception and outcomes. Third, the sample size for analysis of FGID patients was small, and the percentage of patients with IBS alone was much lower than those in western countries; thus, the results might not be generalizable. But it was generally consistent with the illness spectrum in China, especially given consideration to those with overlapping FGIDs. In addition, as presented in the results, about one-third patients in the FIGD group were with high level of depression and anxiety. The psychopathology could have an influence over the mediation effect between the illness perception and other variables. Still, to represent the FGID patients in the real world, those with comorbid mood disorders were not excluded from the sample. Fourth, patients in the TCM and GIM department were consecutively recruited, without excluding those with gastrointestinal complaints and possible FGIDs. However, such overlapping should narrow the difference among six illness groups, instead of compromising the significant difference found currently. Last, but not least, the study was conducted among six groups of patients from four outpatient departments of a Chinese general hospital. Our results should be evaluated with caution concerning patients with different clinical characteristics and cultural backgrounds.

## Conclusion

In all, this study found that (1) the illness perceptions of FGID patients were characterized by broad non-gastrointestinal symptoms, a negative illness representation, and a high number of psychological and culture-specific attributions; (2) illness representation and attribution played a mediating role between the variables of symptomology, alexithymia, and outcomes; and (3) the number of non-gastrointestinal symptoms predicted mental and physical HRQoL, while gastrointestinal symptoms predicted doctor-visit frequency. Our models provide evidence and direction for further research into the relationship between FGID and psychopathology, particularly with regard to dysfunctional illness perceptions and emotion regulation. In addition, the results demonstrate the necessity of both multidisciplinary and individualized management for FGID patients, to provide a chance for improving their quality of life and reducing the overuse of medical resources.

## Data Availability Statements

The raw data supporting the conclusions of this manuscript will be made available by the authors, without undue reservation, to any qualified researcher.

## Ethics Statement

This study was carried out in accordance with the recommendations of Peking Union Medical College Hospital Ethics Committee. All subjects gave written informed consent in accordance with the Declaration of Helsinki. The protocol was approved by the Peking Union Medical College Hospital Ethics Committee.

## Author Contributions

N-nX, JW, and M-yK contributed to the conception and design of the study and wrote the manuscript. XH, TL, L-mZ, YS, and JJ contributed to the data acquisition and interpretation and wrote sections of the manuscript. FF and JW contributed to the data analysis, editing, reviewing, and final approval of the manuscript.

## Conflict of Interest Statement

The submitted work was carried out in the absence of any personal, professional or financial relationships that could potentially be construed as a conflict of interest.

## Abbreviations

GIM, general internal medicine; PU/RE, peptic ulcer/reflux esophagitis.
