# Supplementary material for: Illness Perception of Patients with Functional Gastrointestinal Disorders
Source: Front Psychiatry. 2018 Apr 12;9:122. doi: 10.3389/fpsyt.2018.00122 (PMC5906533; doi:10.3389/fpsyt.2018.00122)
Supplement: Supplementary file 1 [file data_sheet_1.docx]

Supplementary table 1. Clinical diagnosis of patients in the FGID group according to the Rome III diagnostic criteria (n=102)

|  | n (%) |
| --- | --- |
| A. Functional esophageal disorders | 5 (4.9) |
| B. Functional gastroduodenal disorders | 35 (34.3) |
| B1. Functional dyspepsia | 32 (31.4) |
| B2. Belching | 2 (2.0) |
| B3. Nausea and vomiting disorders  B4. Rumination syndrome | 1 (1.0)  0 |
| C. Functional bowel disorders | 24 (23.5) |
| C1. Irritable bowel syndrome | 4 (3.9) |
| C2. Functional bloating | 0 |
| C3. Functional constipation  C4. Functional diarrhea  C5. Unspecified functional bowel disorder | 19 (18.6)  1 (1.0)  0 |
| D. Functional abdominal pain syndrome | 5 (4.9) |
| E. Functional gallbladder disorder | 0 |
| F. Functional anorectal disorders | 2 (2.0) |
| General diagnosis (for those with comorbid FGIDs) |  |
| Functional bowel disorders | 4 (3.9) |
| Functional gastrointestinal disorders | 27 (26.5) |

Supplementary table 2. Clinical diagnosis of patients in the PU/RE group confirmed by the upper gastrointestinal endoscopy (n=95)

|  | n (%) |
| --- | --- |
| Peptic ulcer | 39 (41.1) |
| Stomach ulcer | 14 (14.7) |
| Duodenum ulcer | 25 (26.3) |
| Reflux esophagitis | 56 (58.9) |
| Both types | 3 (3.2) |

Supplementary table 3. Main complaints for doctor-visiting reported by outpatients in the traditional Chinese medicine department (n=102)

|  | n (%) |
| --- | --- |
| **Harmonizing the body/ sub-health status** | 2 1 (20.6) |
| **Symptoms** | 40 (39.2) |
| Gastrointestinal symptoms like dyspepsia, bloating or constipation | 11 (10.8) |
| Skin allergy/ dermatitis/ eczema | 9 (8.8) |
| Irregular menstruation | 5 (4.9) |
| Arthralgia/ heel pain/ low back pain | 5 (4.9) |
| Dizziness/ tinnitus | 4 (3.9) |
| Fatigue/ shortness of breath/ insomnia | 4 (3.9) |
| White and greasy coating on the tone/ liver stagnation | 2 (2.0) |
| **Diseases** | 41 (40.2) |
| Gastrointestinal diseases like atrophic gastritis, fatty liver, colitis | 10 (9.8) |
| Autoimmune disease like systematic lupus erythematous, Sjogren’s syndrome, etc. | 8 (7.8) |
| Metabolic syndrome like diabetes, hypertension, hyperlipidemia, coronary heart disease, etc. | 8 (7.8) |
| Menopausal/ polycystic ovary syndrome | 6 (5.9) |
| Cervical spondylitis/ lumbar disc herniation | 3 (2.9) |
| Skin disease like psoriasis | 2 (2.0) |
| Chronic glomerulonephritis | 2 (2.0) |
| Thyroid nodules | 1 (1.0) |
| Lung cancer | 1(1.0) |

Supplementary table 4. Main complaints for doctor-visiting reported by outpatients in the western general internal medicine department (n=100)

|  | n (%) |
| --- | --- |
| **Symptoms** | 46 (46.0) |
| Joint pain/ joint swelling/ edema/ ascites | 13 (13.0) |
| Stomach discomfort/ abdominal pain/ hepatic discomfort | 12 (12.0) |
| Unexplained fever | 8 (8.0) |
| Chest pain/ short of breath | 5 (5.0) |
| Cough/ sore throat | 4 (4.0) |
| Dizziness/ headache | 2 (2.0) |
| Weight loss | 1 (1.0) |
| Hematuria | 1 (1.0) |
| **Diseases** | 54 (54.0) |
| Autoimmune diseases like Systemic lupus erythematosus, Sjogren's syndrome, rheumatoid arthritis, etc. | 29 (29.0) |
| Metabolic syndrome like diabetes, hypertension, hyperlipidemia, cerebrovascular disease, etc. | 12 (12.0) |
| Pneumonia/ upper respiratory tract infection | 4 (4.0) |
| Lymphoma | 2 (2.0) |
| Thyroid cancer/ subacute thyroiditis | 2 (2.0) |
| [Renal](http://cn.bing.com/dict/search?q=renal&FORM=BDVSP6&mkt=zh-cn) [calculus](http://cn.bing.com/dict/search?q=calculus&FORM=BDVSP6&mkt=zh-cn)/ nephritis | 2 (2.0) |
| Osteoarthritis | 1 (1.0) |
| [Hemochromatosis](http://cn.bing.com/dict/search?q=hemochromatosis&FORM=BDVSP6&mkt=zh-cn) | 1 (1.0) |
| Urticaria/ vitiligo | 1 (1.0) |

Supplementary table 5. Direct, indirect, and fit indices of structural equation models in overall patients (n = 600)

|  | | MCS | PCS | | Doctor visits |
| --- | --- | --- | --- | --- | --- |
| Standardized effect | |  |  | |  |
| TAS | Direct | -.085 | - | | - |
|  | Indirect | -.045 | - | | - |
| Non-gastrointestinal symptoms | Direct | -.233 | -.265 | | - |
|  | Indirect | -.231 | -.049 | | - |
| Gastrointestinal  symptoms | Direct | - | - | | .086 |
|  | Indirect | - | - | | .047 |
| Model fit | χ² (d.f.) | 1.9 (3) | 5.4 (4) | 1.7 (3) | |
|  | RMSEA  (95%CI) | .000 (.000, .058) | .025  (.000, .070) | .000  (.000, .056) | |
|  | CFI | 1.000 | .997 | 1.000 | |
| Total variance explained (%) | | 43.1 | 29.1 | 17.7 | |
